# Pancreatic arteriovenous malformation: A case report

**DOI:** 10.1097/MD.0000000000042427

**Published:** 2025-05-09

**Authors:** Jianping Liu, Xiaojun Xue, Songrong Lin, Liming Yang, Song Zhou

**Affiliations:** a Department of General Surgery, Dongnan Hospital of Xiamen University, School of Medicine, Xiamen University, Zhangzhou, China; b Department of Pathology, Dongnan Hospital of Xiamen University, School of Medicine, Xiamen University, Zhangzhou, China.

**Keywords:** acute pancreatitis, distal pancreatectomy combined with splenectomy, pancreatic arteriovenous malformation

## Abstract

**Rationale:**

Pancreatic arteriovenous malformation (PAVM) is predominantly caused by congenital factors and is an extremely rare vascular anomaly. The number of documented and reported cases in the literature remains very low.

**Patient concerns:**

We report a case of a 37-year-old male who was admitted to the hospital due to acute abdominal pain.

**Diagnoses:**

Computed tomography revealed an arteriovenous malformation in the tail of the pancreas and acute pancreatitis.

**Interventions:**

The patient underwent a distal pancreatectomy combined with splenectomy.

**Outcomes:**

Histopathological examination confirmed the presence of an arteriovenous malformation in the tail of the pancreas along with acute pancreatitis. We believe that the acute pancreatitis was induced by the PAVM. The patient was discharged successfully and remained symptom-free during follow-up.

**Lessons:**

PAVM is a rare vascular abnormality occurring in the pancreas. Clinical manifestations can include gastrointestinal bleeding, intra-abdominal hemorrhage, pancreatitis, portal hypertension, and pancreatic pseudocyst. Diagnosis can be confirmed through ultrasound, contrast-enhanced computed tomography, magnetic resonance imaging, and digital subtraction angiography. Surgical treatment is an effective approach for symptomatic PAVM.

## 1. Introduction

Pancreatic arteriovenous malformation (PAVM) is a rare vascular anomaly of the pancreas, accounting for approximately 0.9% of gastrointestinal arteriovenous malformations (AVMs).^[1]^ In recent years, advancements in imaging technology have led to an increase in reported cases. While some PAVMs are asymptomatic, most cases present with various symptoms such as abdominal pain, acute pancreatitis, or gastrointestinal bleeding.^[1,2]^ This study reports a case of PAVM complicated by acute pancreatitis, successfully treated through surgical resection. By reviewing the clinical course of this case, we aim to provide insights into the diagnosis and treatment of PAVM.

## 2. Case presentation

**Figure 1.**
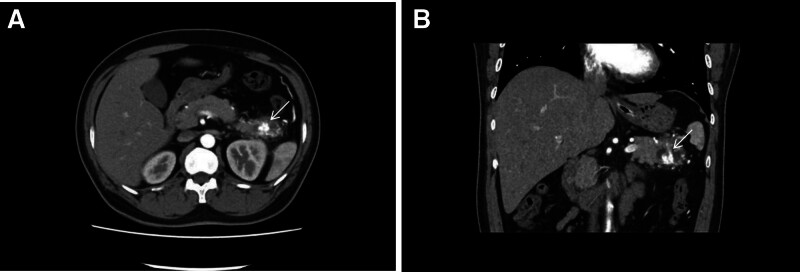
(A and B) Arterial phase-enhanced computed tomography shows a nodular hyperdense lesion in the tail of the pancreas.

**Figure 2.**
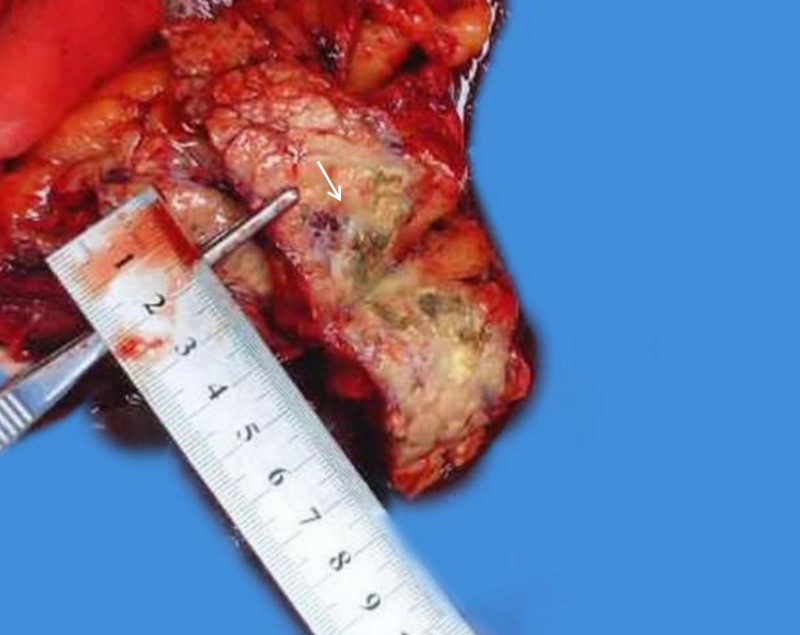
The arrow indicates an area formed by dilated arteriovenous vessels, accompanied by thrombosis.

A 37-year-old male presented to the outpatient clinic with left-sided abdominal pain lasting more than 10 days. The pain did not improve with oral medication, and he was admitted for further treatment. Laboratory tests revealed a white blood cell count of 11.86 × 10^9^/L (reference range 3.50–9.50 × 10^9^/L), serum amylase of 157 U/L, and serum lipase of 203 U/L. Enhanced contrast computed tomography (CT) showed an enlarged pancreatic tail with tortuous and widened arterial structures, blurred marginal fat, and multiple areas of low-density exudative changes around the pancreas, consistent with the appearance of a PAVM (Fig. 1A and B). Due to the patient’s concurrent acute pancreatitis, the lesion at the pancreatic tail was densely adherent to the splenic artery and vein, making dissection impossible. As a result, we performed an open resection of the pancreatic tail along with splenectomy. During the procedure, we observed that the blood vessels supplying the pancreatic tail, arising from the splenic artery, omental vessels, and vessels adjacent to the descending colon, were dilated and tortuous. The pancreatic tissue was relatively firm and tightly adherent to surrounding structures. The surgery proceeded smoothly, lasting 3 hours and 41 minutes, with an estimated blood loss of 300 mL. Postoperative histopathology revealed a firm area in the pancreatic tail, showing liquefactive necrosis and proliferative, dilated blood vessels. The vessel walls were thickened, and there was congestion within the lumens, consistent with acute pancreatitis associated with changes due to AVM (Fig. 2). Postoperative complications included: Localized atelectasis in both lower lungs; bilateral lower lobe pneumonia with a small amount of left-sided pleural effusion; encapsulated fluid collection at the surgical site; elevated platelet count. There were no complications such as hemorrhage or pancreatic leakage. The patient was managed symptomatically and recovered fully. The postoperative course was uneventful, and the patient was discharged 13 days after surgery. A follow-up CT scan on August 25, 2023, showed postoperative changes in the surgical area, with no evidence of AVMs elsewhere in the body.

## 3. Discussion

PAVM is a rare disease. Since its first description by Halpern in 1968,^[3]^ the number of cases reported in the literature remains low. Due to the presence of asymptomatic patients, the actual prevalence is unknown. The formation of PAVM is thought to be related to the loss of sphincter function in the walls of the supplying arteries, leading to direct blood flow from the arterial system into the portal venous system.^[4,5]^ The causes of PAVM include congenital and acquired factors. Congenital cases are due to the abnormal development of the primitive vascular plexus during the embryonic period, accounting for about 90%, while acquired cases are often secondary to malignant tumors, traumatic injuries, or pancreatitis, accounting for only 10%.^[4]^ The patient in this case presented with the disease for the first time, with no history of tumors, trauma, or pancreatitis, suggesting that their PAVM might be congenital.

The clinical symptoms of PAVM vary among different patients, including gastrointestinal bleeding, epigastric pain, and portal hypertension. There are also reports of pancreatitis caused by PAVM.^[6]^ These clinical symptoms are usually related to the location of the occurrence. In pancreatic head AVM, bleeding is the most common manifestation, while in pancreatic body and tail AVM, pancreatitis is more common. This patient developed pancreatitis in the tail of the pancreas, consistent with the reports^.[7]^ The patient’s abdominal pain was caused by pancreatitis, and the pain was relieved after treatment targeting pancreatitis.

Cases associating PAVM with pancreatitis are rare, and the relationship between the 2 can be reciprocal. The mechanism by which PAVM causes pancreatitis is not well understood but may be related to several factors: bleeding from the PAVM involving the pancreatic duct; ischemic damage caused by the PAVM stealing blood flow from the surrounding normal pancreatic parenchyma; compression of the pancreatic duct by the PAVM.^[8]^ Additionally, cases where pancreatitis leads to PAVM show that pancreatitis can cause vascular damage due to the action of pancreatic enzymes, including thrombosis or destruction of intrapancreatic and peripancreatic vessels, resulting in a pancreatic arteriovenous fistula. We believe that the patient’s pancreatitis was secondary to PAVM because pathological examination of the resected specimen showed an area of liquefactive necrosis in the pancreatic tail, likely due to ischemia caused by the PAVM. No intraductal hemorrhage was observed in the specimen, and CT did not show pancreatic duct dilation.

Due to the rarity of cases and nonspecific symptoms, the diagnosis of PAVM often relies on imaging studies, including CT, magnetic resonance imaging, color Doppler ultrasound, and angiography. On CT, PAVM appears as nodular staining in the arterial phase and early enhancement in the portal venous phase.^[9]^ In our patient, enhanced contrast CT revealed these features. Considering the good diagnostic capability and noninvasive nature of enhanced contrast CT, and the invasive nature of angiography, our patient did not undergo angiography.

For symptomatic patients, various treatment options are currently available, including surgical and nonsurgical approaches such as transarterial embolization, radiotherapy, and transjugular intrahepatic portosystemic shunt.^[10]^ Surgical resection of the PAVM lesion can achieve a cure and prevent long-term complications. This patient presented with symptoms of PAVM for the first time, and preoperative examinations did not indicate portal hypertension. To avoid recurrent pancreatitis symptoms and long-term complications, we recommended surgical treatment. During the surgery, significant adhesion between the lesion and the spleen was observed, so we performed a distal pancreatectomy combined with splenectomy. The patient had a smooth postoperative recovery, and follow-up showed no recurrence of the original symptoms, demonstrating the effectiveness of surgery for this condition.

Due to the multiple arterial supplies to PAVMs, complete embolization or ligation is challenging. Therefore, transarterial embolization is typically used as an alternative treatment for patients with high surgical risk, those who refuse surgery, or for controlling acute gastrointestinal bleeding.^[11]^ Additionally, transjugular intrahepatic portosystemic shunt and radiotherapy are viable treatment options when the surgical risk is high.^[1]^

## 4. Conclusion

Previous cases have shown that when symptoms such as pancreatitis or bleeding occur, surgery is the recommended treatment. Our experience also supports that surgery is an effective treatment method. The surgical approach should be determined based on the lesion location, and regular follow-up is necessary postoperatively.

## Author contributions

**Conceptualization:** Xiaojun Xue.

**Data curation:** Liming Yang.

**Funding acquisition:** Song Zhou.

**Investigation:** Xiaojun Xue, Liming Yang.

**Supervision:** Song Zhou.

**Validation:** Song Zhou.

**Visualization:** Song Zhou.

**Writing – original draft:** Jianping Liu, Songrong Lin.
